# Vision and Hyper-Responsiveness in Migraine

**DOI:** 10.3390/vision3040062

**Published:** 2019-11-11

**Authors:** Amelia Aldrich, Paul Hibbard, Arnold Wilkins

**Affiliations:** Department of Psychology, University of Essex, Wivenhoe Park, Essex, Colchester CO4 3SQ, UK; aaldri@essex.ac.uk (A.A.); phibbard@essex.ac.uk (P.H.)

**Keywords:** migraine aura, contrast discrimination, cortical hyperexcitability

## Abstract

We investigated contrast processing in relation to visual comfort from coloured light in individuals with migraine. In Experiment 1, 24 individuals who experienced migraine with aura (MA), 15 migraine without aura (MO), and 23 healthy controls, identified which of four patterns, one in each quadrant, had the greatest contrast. Although there were no significant differences between groups, contrast discrimination was superior in the visual field affected by aura in all eight participants in whom the aura was consistently lateralised. In Experiment 2, 20 participants without aura and 20 controls selected comfortable light with a chromaticity close to the daylight (Planckian) locus, whilst 20 individuals with aura chose more strongly saturated colours, mostly distant from the locus. In Experiment 3, nine participants with consistently unilateral aura undertook the contrast discrimination task wearing (a) lenses that provided a comfortable colour of light and (b) grey lenses of similar transmission. With grey lenses, seven of the nine individuals with unilateral aura showed a superior performance in the affected field, as before. With lenses providing a comfortable colour, however, the performance was relatively poor for the nine individuals with unilateral aura, but not for the 10 controls. This was the case in both visual fields. The cortical hyper-responsiveness with which migraine is associated may improve the perception of contrast. The perception is poorer (and more normal) with ophthalmic lenses having a comfortable colour.

## 1. Introduction

Migraine is a severe form of headache associated with sensitivity to bright light and strong patterns [1,2]. Migraine affects vision, decreasing contrast sensitivity [3,4,5,6], although a deficit is not invariably found [7,8] and the visual performance is sometimes superior in individuals with migraine [9,10,11]. There is convergent but controversial evidence that in migraine, the cortex is hyperexcitable or at least hyper-responsive [12,13,14], but little is known about any influence of such hyper-responsiveness on visual function. Ophthalmic tints selected by individuals to provide a comfortable colour of light have been shown to reduce the hyper-BOLD response [15] and here, we explore the consequences of colour for visual function. We show that contrast discrimination is superior in the visual field affected by aura and that this superiority is reduced by a comfortable colour of tint.

## 2. Experiment 1: Contrast Discrimination

### 1.1. Participants

Forty-nine females and 13 males (aged 18–58) were recruited from students and staff at the University of Essex: 23 headache-free controls, 15 migraine without aura (MO) and 24 with visual aura (MA). Participants with migraine fulfilled the International Headache Society’s ICHD III criteria [16] for migraine with or without aura and provided data on the time since the last migraine attack and duration of disease (see Table S1 Supplementary Materials). Participants in the MA group were asked to draw their migraine aura. From these drawings, individuals whose aura was confined to one visual field were identified. Control participants had never experienced a migraine. The mean number of days since the previous attack of migraine was 54.5 (range = 2–365) for the MO group, 17.6 (range = 2–93) for the MA group with bilateral visual aura and 161 (range = 2–730) for those with unilateral aura. The mean duration of disease was 5.2 years (range = 2–11) for the MO group, 6.06 (range = 2–15) for the MA group with bilateral aura and 17.1 (range = 2–50) for the MA group with unilateral aura. 

### 1.2. Procedure

**Figure 1:**
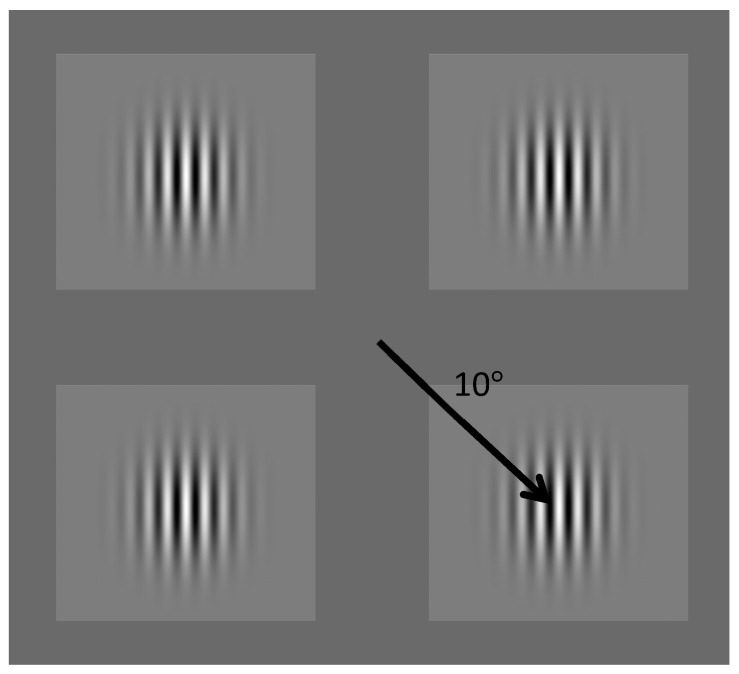
Schematic diagram of the four gratings with centres 10 degrees from fixation.

Stimuli were presented on a Sony Multiscan 500 Trinitron CRT monitor using a Windows PC. The screen had a resolution of 1280 × 1024 pixels and a refresh rate of 120 Hz running custom Matlab (Mathworks R_2014b) software using the Psychophysics Toolbox extensions [17,18]. The luminance resolution of the screen was controlled by a 14-bit Datapixx system (VPixx Technologies, Quebec, Canada). A ResponsePixx (VPixx Technologies, Quebec, QC, Canada) button box was used for participant responses. The screen was calibrated using a photometer (Minolta, Tokyo, Japan) and had a mean luminance of 27.5 cd/m^2^. Stimuli were vertical gratings with contrast that tapered towards the edges (Gabors), with a spatial frequency of 2.6 cpd and a standard deviation of 59 arcmin subtending 30 arcmin (see Figure 1).

A four-alternative forced choice (4AFC) paradigm was utilised with three separate, interleaved staircase procedures (three-up, one-down; one-up, one-down; one-up, three-down), each consisting of 40 trials. For each staircase, the contrast of the ‘target’ pattern was incremented and decremented in steps of 0.05 Michelson contrast for the first 20 trials, and in steps of 0.025 for the remaining 20 trials. Individuals were seated 0.6 m from the screen in a dark room.

Participants were examined when wearing any habitual refraction. They completed eight blocks of trials in total, which were split into two blocks of four. Four blocks of trials had a pedestal contrast (i.e., the contrast against which the contrast was incremented) of 10% and the remaining four blocks had a pedestal contrast of 50%. Individuals were instructed to view a central fixation cross, which appeared on the screen for 100 ms during each presentation lasting 142 ms (17 refresh cycles). The two blocks of trials were counterbalanced between participants to minimise any practice effects. Upon finishing the first block of trials, participants completed a questionnaire regarding their experience of migraine. 

The three experiments reported herein were approved by the University of Essex ethics committee and adhere to the tenets of the Declaration of Helsinki. All participants gave written informed consent after a full explanation of the methods.

## 3. Results

A cumulative Gaussian curve was fitted to the data using Matlab and the Palamedes toolbox, with alpha (contrast threshold) and beta (slope) as free parameters, using a maximum likelihood fit. This was used to calculate each participant’s 62.5% contrast discrimination threshold for the 10% and 50% contrast conditions, as appropriate for four-alternative forced choice. The contrast discrimination thresholds for all observers for 10% and 50% contrast were subjected to a 3 (group) × 2 (contrast level) analysis of variance. The group was the between-subjects factor and the low and high pedestal conditions the within-subjects factor. The contrast discrimination threshold was significantly higher in the 50% condition (*F* (1, 59) = 36.91, *p* < 0.001, η^2^ = 0.38). There was no main effect of the group (*F* (2, 59) = 0.94, *p* = 0.395, η_p_^2^ = 0.031) and no significant interaction between the contrast and group (*F* (2, 59) = 0.07, *p* = 0.936, η^2^ = 0.0014). 

**Figure 2:**
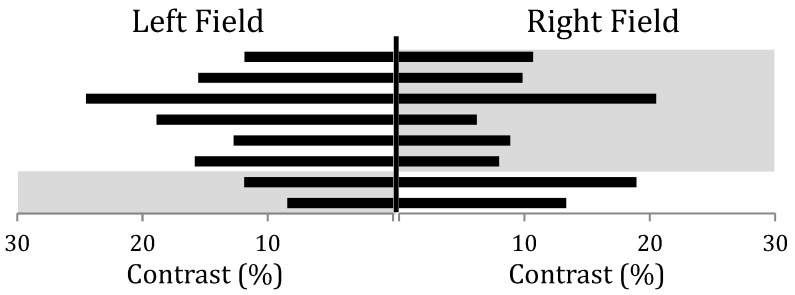
The average contrast discrimination threshold for the eight participants who experienced lateralised visual aura. The shaded areas group the six who experienced aura in the right field and the two who experienced aura in the left field.

Eight of the 24 participants in the MA group experienced a consistently unilateral visual aura. The contrast threshold was calculated separately for each quadrant of the screen. A psychometric curve was fitted to all of the data from the left and right hemifields separately. The pedestal contrast was subtracted from the threshold in order to obtain the increment necessary for an individual to detect the target. The increment was generally smaller in the affected field for both the 10% and 50% pedestal conditions. For all eight participants with unilateral aura, the contrast threshold was lower in the field affected by aura when averaged across conditions (see Figure 2). Paired-sample t-tests revealed significantly lower thresholds in the affected field for the 10% and 50% conditions (*t*(7) = 2.94, *p* = 0.022 and *t*(7) = 3.91, *p* = 0.006 respectively).

## 4. Experiment 2: Comfortable Chromaticity

### 4.1. Participants

A further 49 female and 11 male students and staff (aged 18–55) from the University of Essex completed a computer-administered questionnaire, based on the International Headache Society’s criteria for migraine, both with and without aura. The first 15 individuals who fulfilled the criteria were selected for the MA group, MO group and headache-free groups. The remaining five individuals in each group were recruited via an internal distribution list and completed the questionnaire on the day of testing. Individuals in the control group reported that they had never experienced a migraine. The mean age of the control, MO and MA groups was 19.7, 20.7, and 23.2 years, respectively. The mean number of weeks since the previous attack of migraine was 2.5 (range = 0.5–10) for the MA and 3.1 (range = 0.5–10) for the MO group. The mean duration of disease was 3.4 years (range = 1–7) for the MO group and 6.6 (range = 2–30) for the MA group. All the MA group experienced visual aura. See Table S2 in Supplementary Materials for a summary of participants’ details.

### 4.2. Procedure

A Intuitive Colorimeter Mk.II (Cerium Visual Technologies, Tenterden, Kent) was used to identify a comfortable and uncomfortable colour of light. The procedure for colorimetry [19] compared 12 moderately saturated colours with white at a constant luminance of about 30 cd·m^−2^ and then iteratively manipulated the hue and saturation of those shortlisted as preferable, allowing for adaptation to the colours, as described in the Intuitive Colorimeter manual. The 60 participants were individually tested. None reported colour vision deficits. A refractive correction was used by 21 participants. All underwent a colorimetry examination to identify a comfortable colour of illumination. The most uncomfortable colour was selected from the reports of discomfort during the initial presentation of 12 moderately saturated colours, so as to avoid any additional discomfort. 

## 5. Results

**Figure 3:**
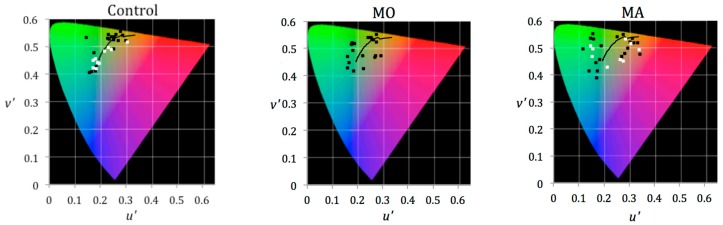
Commission Internationale de l’Eclairage 1976 Uniform Chromaticity Scale diagrams showing the comfortable colours selected by each group in Experiment 2 (black points) and Experiment 3 (white points) with the Planckian locus superimposed.

The chromaticities of the colours chosen as comfortable are presented in the CIE 1976 UCS diagram in Figure 3, which also shows the data from Experiment 3 (individuals who experienced MO were not sampled in Experiment 3). 

The difference in chromaticity between each participant’s comfortable colour and the nearest point on the daylight locus was calculated [20] and is shown in Table 1. A one-way between-subjects ANOVA was conducted to examine the effect of participant group on the distance from comfortable colours to the daylight locus. This revealed a significant effect of group (*F*(2, 57) = 4.259, *p* = 0.019). Planned comparisons with a Bonferroni correction revealed a significant difference between the control and MA groups (adjusted *p* = 0.028) and no significant difference between the control and MO groups (*p* = 1.000) or the MA and MO groups (*p* = 0.071). 

## 6. Experiment 3: Contrast Discrimination and the Effects of Colour

### 6.1. Participants

Further participants were recruited from students and staff of the University of Essex. One of the MA participants had earlier participated in the contrast discrimination task described in Experiment 1. Individuals in the migraine with aura (MA) group experienced a consistently unilateral visual aura. Nineteen participants were recruited for this study: 9 MA (4 who experienced aura in the left visual field and 5 who experienced aura in the right visual field) and 10 migraine-free control participants. Individuals in the migraine group fulfilled the International Headache Society’s ICHD III criteria for migraine with aura. Participants in the control group had never experienced a migraine. No colour-vision anomalies were identified by Ishihara tests of colour vision (Kanehara Shuppan Co., Ltd., Tokyo, Japan) and the City University Colour Vision Test 3rd Edition (Keeler, Windsor, England). The mean number of days since the previous attack of migraine was 9.3 (range = 2–21) for those who experienced aura in the left visual field and 43.8 (range = 2–112) for those who experienced aura in the right field. The mean duration of disease was 8.5 years (range = 3–18) for those who experienced aura in the left visual field and 22.0 years (range = 10–30) for those who experienced aura in the right field. See Table S3 in Supplementary Materials for participants’ details.

### 6.2. Procedure

The contrast discrimination stimuli and apparatus from Experiment 2 were used in Experiment 3, although only the stimuli with a 50% pedestal contrast were presented. The procedure from Experiment 1 was used. As in Experiment 2, an Intuitive Colorimeter Mk.2 (Cerium Visual Technologies, Tenterden, Kent, UK) was used to identify a colour of illumination that was comfortable for viewing text. Each participant completed the contrast discrimination task wearing two sets of coloured lenses in counterbalanced order. One set of lenses matched the colour chosen as comfortable in the Intuitive Colorimeter, and the second set were grey lenses of closely matched transmission. The lenses were from the trial set of filters that accompany the Intuitive Colorimeter (Cerium Visual Technologies, Tenterden, Kent, UK) and have spectral transmissions given by Wilkins et al. [21]. The lenses were placed in a holder and secured with elastic around the participant’s head. Trials were presented in two blocks of four with a pedestal contrast of 50% only. Individuals wore the first set of lenses to complete the first four blocks of trials. Participants then completed the last four blocks of trials wearing the second set of lenses.

## 7. Results

**Figure 4:**
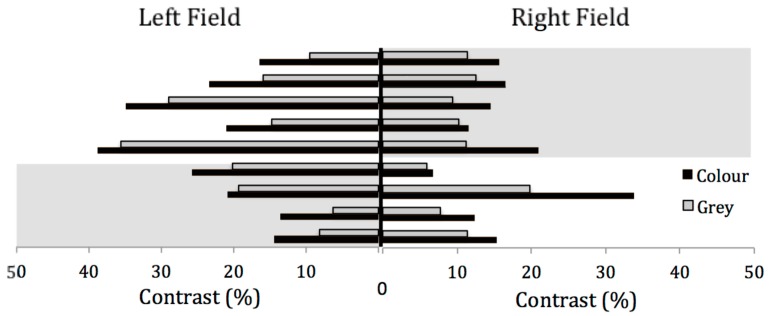
Contrast discrimination results for Experiment 3. Black bars illustrate the performance when lenses of a comfortable colour were worn and grey bars illustrate the performance when control grey lenses were worn. The shaded areas indicate the side of aura, as in Figure 2.

Figure 4 shows the contrast discrimination threshold when participants were wearing coloured lenses of a comfortable colour and when control grey lenses were worn. In Experiment 3, seven of the nine participants had a lower contrast discrimination threshold in the field affected by lateralised visual aura, although the difference between fields was not significant.

Differences between contrast discrimination for the unilateral aura and control groups were examined. A conservative approach was used to compare thresholds with the grey lenses for the affected field of the unilateral aura group with similar thresholds for the right visual field of the controls, which were significantly lower (more sensitive) than the left (*t*(22) = 4.303, *p* < 0.001). The contrast discrimination thresholds in the affected field of the unilateral aura group were significantly lower than those of the control group in the right visual field (*t*(29) = 2.127, *p* = 0.042). This analysis and that of Experiment 1 indicates that the performance may be supra-normal in the unilateral aura group, compared to controls, but the sample size is small and this complex effect will require further examination. 

**Figure 5:**
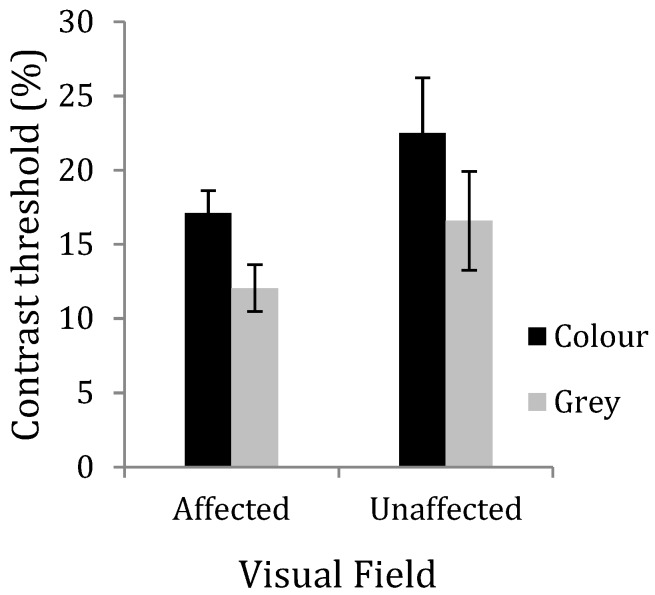
Average contrast discrimination threshold for the migraine with aura (MA) group in Experiment 3, shown separately for both the visual field that was affected by aura and the field that was unaffected. Error bars denote 1 standard error of the mean.

MA group results were organized according to the field affected by visual migraine aura and the field that was unaffected by aura (see Figure 5). It would be anticipated that the control grey lenses would have little or no effect on the contrast discrimination threshold and that the threshold would be lower in the field affected by aura, as in Experiment 1. A 2 × 2 within-subjects ANOVA was conducted for the MA group, with the visual field (affected and unaffected by aura) and colour of the lenses as the main effects. There was no effect of visual field (F(1, 8) = 1.74, *p* = 0.22, η^2^ = 0.18), but there was a significant effect of colour (F(1, 8) = 142.8, *p* < 0.001, η^2^ = 0.94). The interaction between colour and field was not significant (F(1, 8) = 0.20, *p* = 0.67, η^2^ = 0.02). The contrast discrimination threshold was not significantly lower for the MA group in the affected field compared to the unaffected field when wearing the grey lenses (*t*(8) = 1.19, *p* = 0.27). However, when the difference between the coloured and grey lenses was examined in the affected field, coloured lenses increased the contrast discrimination threshold (*t*(8) = 5.71, *p* < 0.001). The coloured lenses also significantly raised the contrast discrimination threshold in the unaffected field (*t*(8) = 4.819, *p* < 0.001) (see Figure 5 and Table 2).

**Figure 6:**
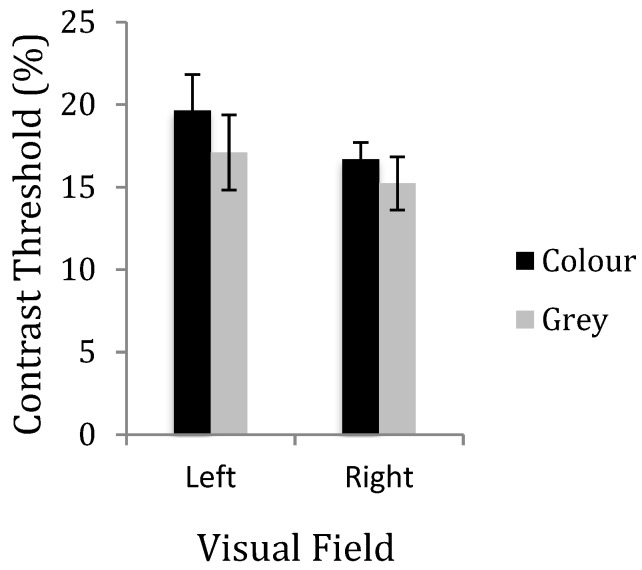
Mean contrast discrimination threshold for the left and right visual fields in the control group.

A 2 × 2 within-subjects ANOVA was conducted for the control group, with the visual field and colour of the lenses as the main effects. There was no significant effect of visual field on the contrast discrimination threshold in the left and right visual fields (*F*(1, 9) = 1.664, *p* = 0.229, η^2^ = 0.156). For the controls, there was no effect of colour of the lenses (*F*(1, 9) = 4.839, *p* = 0.055, η^2^ = 0.349) and no interaction (F(1, 9) = 0.850, *p* = 0.381, η^2^ = 0.086). The contrast threshold when wearing the ‘active’ and grey lenses was compared for the left visual field (*t*(9) = 2.26, *p* = 0.50) and for the right field (*t*(9) = 1.40, *p* = 0.196) (see Figure 6).

The contrast discrimination threshold obtained in the left and right visual fields was therefore averaged for post hoc comparisons of controls and the MA group. An independent sample t-test was conducted to establish whether the transmission of the coloured lenses, and that of the grey, differed between groups. The transmission of the grey lenses was significantly greater in the control group (i.e., the lenses were less saturated) (*t*(17) = 2.97, *p* = 0.009). The transmission of the coloured lenses was also significantly greater for the control group (*t*(17) = 2.91, *p* = 0.010). Participants in the MA group chose lenses of a greater saturation.

For the control group, the chromaticities of the lenses chosen as comfortable lie close to the Planckian locus. The distance of the data point closest to the locus was calculated. An independent sample t-test revealed that the colours of the lenses selected by controls were significantly closer to the daylight locus (*t*(9.09) = 3.28, *p* = 0.009).

The chromaticities of colours chosen as comfortable during Experiment 2 were added to the current results to further examine the proximity to the Planckian locus. Again, the colours chosen by the control group were significantly closer to the Planckian locus (*t*(31.59) = 2.35, *p* = 0.025). Additionally, the corrected significance level was reported because the variance was unequal. The chromaticities of the various forms of indoor lighting lie close to the Planckian locus. Although the colours ranged from yellow to blue, it is possible that individuals in the control group chose lenses of colour similar to that of the lighting they experience every day.

## 8. Discussion

The causes of migraine are complex and poorly understood. Here, we are concerned with the visual differences with which migraine is associated. These may or may not be associated with the triggering of attacks. Cortical hyper-responsiveness or excitability, as proposed by many [5,6,7,8], provides one possible explanation for the visual differences.

We have shown superior contrast discrimination in the visual field affected by lateralised visual aura in migraine, consistent with an effect of hyper-responsiveness on visual function. The colour chosen as comfortable for viewing text reduces the abnormal contrast discrimination, consistent with the effect of colour in reducing the hyper-BOLD response to patterns in migraine [14].

If the differences in responsiveness are due to hyperexcitability, the individual differences with respect to the choice of a comfortable colour may reflect differences in a cortical locus of the hyperexcitability (hyperexcitability is unlikely to be uniform, as demonstrated by the selective response to pattern orientation in patients with pattern-sensitive epilepsy [22]). In the visual area, V2 cells are colour-coded and arranged on the cortical surface topographically, according to perceptual colour [23]. We hypothesise that coloured light re-distributes the cortical activity induced by the visual stimulus in such a way as to avoid local areas of hyperexcitability.

The results of Experiments 2 and 3 suggest that

(1) Those for whom colour is of no benefit choose light of familiar colours to which they are habitually exposed. Perhaps those patients who are most likely to benefit from tinted lenses choose colours away from the Planckian locus,
(2) Individuals with migraine are rarely exposed to light of a colour they find comfortable, which may have consequences for photophobia. Noseda et al. [24] have proposed a role for the intrinsically photosensitive retinal ganglion cells (ipRGC) in photophobia. The cells are not themselves sensitive to patterns of the kind used here, but the ipRGC cells may indirectly contribute to contrast sensitivity [25]. The ipRGC are melanopic, with the spectral sensitivity being greatest at short wavelengths. The distribution of selected chromaticities shows no preference for short-wavelength light.

The individuals sampled in the three experiments reported here were from the general population and not tertiary referrals to neurology clinics. Participants in the migraine groups may therefore be less impaired than those reported in the literature.

## Figures and Tables

**Table 1:** Mean (SD) distance from the Planckian locus for each group.

Group	Mean	SD
Control	0.00107	(0.00158)
MO	0.00129	(0.00114)
MA	0.00265	(0.00256)

**Table 2:** The results of paired-sample t-tests for the MA group based on the visual field affected by aura and the colour of the lenses.

	Affected Field Coloured Lens	Unaffected Field Grey Lens
**Unaffected field Coloured lens**	*p* = 0.212	*p* = 0.001
**Affected field**	*p* = 0.001	*p* = 0.267
**Grey lens**		

## Supplementary Materials

The following are available online at https://www.mdpi.com/2411-5150/3/4/62/s1: Table S1: Experiment 1, Table S2: Experiment 2, Table S3: Experiment 3.
