# Supplementary material for: Vision and Hyper-Responsiveness in Migraine
Source: Vision (Basel). 2019 Nov 11;3(4):62. doi: 10.3390/vision3040062 (PMC6969908; doi:10.3390/vision3040062)
Supplement: Supplementary file 1 [file vision-03-00062-s001.pdf]

Table 1

Experiment 1

	C	MO	MA	
**N**	23	15	24	
**Age Mean** (SD)	22.4 (4.7)	22.4 (3.1)	27.3 (11.8)	
**Age range**	19-38	18-28	18-58	
**Using refractive correction**	11	10	12	
			**Bilateral aura**	**Unilateral aura** (N = 8)
**Mean days since last attack** (SD)	N/A	54.5 (100)	17.6 (31)	161 (258)
**Mean (SD) duration of disease** (years)	N/A	5.2 (2.9)	6.06 (3.7)	17.1 (17.6)

Table 2

Experiment 2

	Control	MO	MA
**M/F**	7 M 13 F	1 M 19 F	3 M 17 F
**Age range**	18-21	18-24	18-55
**Mean age** (SD)	19.7 (0.9)	20.7 (2.0)	23.2 (9.5)
**Using refractive Correction**	7	6	8
**Mean (SD) duration of disease** (years)	N/A	3.4 (2.0)	6.6 (7.2)
**Mean weeks since last attack** (SD)	N/A	3.1 (2.5)	2.5 (2.4)
**Prophylactic medication**	N/A	1	3

Table 3

Experiment 3

	**C**	**MA Aura Left Field**	**MA Aura Right Field**
**N**	10	4	5
**Mean age** (SD)	19.7 (1.70)	21.5 (5.07)	34.2 (9.81)
**Age Range**	18-23	18-29	22-48
**Using refractive correction**	5	3	4
**Mean days since last attack** (SD)	N/A	9.3 (8.2)	43.8 (42.7)
**Mean (SD) duration of disease** (years)	N/A	8.5 (6.9)	22.0 (7.6)
